# Suberoylanilide Hydroxamic Acid Analogs with Heteroaryl Amide Group and Different Chain Length: Synthesis and Effect on Histone Deacetylase

**DOI:** 10.3390/molecules29010238

**Published:** 2024-01-01

**Authors:** Gabriele Micheletti, Carla Boga, Giacomo Drius, Silvia Bordoni, Natalia Calonghi

**Affiliations:** 1 Department of Industrial Chemistry ‘Toso Montanari’, Alma Mater Studiorum, Università di Bologna, Viale Del Risorgimento 4, 40136 Bologna, Italy; giacomo.drius2@unibo.it (G.D.); silvia.bordoni@unibo.it (S.B.); 2 Department of Pharmacy and Biotechnology, University of Bologna, 40127 Bologna, Italy

**Keywords:** HDAC, SAHA, azaheterocycles, synthesis, anticancer

## Abstract

This review covers the last 25 years of the literature on analogs of suberoylanilide hydroxamic acid (SAHA, known also as vorinostat) acting as an HDAC inhibitor. In particular, the topic has been focused on the synthesis and biological activity of compounds where the phenyl group (the surface recognition moiety, CAP) of SAHA has been replaced by an azaheterocycle through a direct bond with amide nitrogen atom, and the methylene chain in the linker region is of variable length. Most of the compounds displayed good to excellent inhibitory activity against HDACs and in many cases showed antiproliferative activity against human cancer cell lines.

## 1. Introduction

Histone deacetylases (HDACs) and histone acetyltransferases (HATs) catalyze, respectively, deacetylation and acetylation of specific lysine residues situated on the amino-terminal tails of histone proteins. These enzymes play a key role in gene transcription [1] since acetylation is associated with an open chromatin configuration resulting in enhancing transcription [2] whereas the deacetylation process induces condensed and transcriptionally inactive heterochromatin [3].

Normally, it exists as a balance between histone acetylation and deacetylation in normal cells; however, it has also demonstrated that these two enzymes are not only involved in the regulation of chromatin structure and gene expression, but they can also regulate cell cycle progression and carcinogenic processes [4]. Inhibition of HDACs can lead to cell differentiation, apoptosis, and cell cycle arrest both in several cancer cell lines and in vivo, thus making HDAC inhibitors (HDACIs) a very important class of anticancer agents [5,6]. Besides their anticancer effects, some HDACIs also exhibit valuable neuroprotective properties in brain injuries such as stroke [7] and ischemia [8]. Further, some studies have reported the potential of HDACIs to treat chronic neurological disorders such as amyotrophic lateral sclerosis [9] and Alzheimer’s disease [10].

The common classification of HDACs is based on a molecular phylogenetic analysis of the primary structure. They are grouped (based on homology to yeast enzymes [11]) in distinct classes: class I (HDAC1, HDAC2, HDAC3, and HDAC8), class IIa (HDAC4, HDAC5, HDAC7, and HDAC9), class IIb (HDAC6 and HDAC10), and class IV (HDAC11); these classes contain zinc-dependent domains. The class III belongs to a structurally and mechanistically distinct class of NAD^+^-dependent hydrolases (sirtuins, Sirt1–Sirt7) [12].

HDAC inhibitors have been classically structurally grouped into four classes: hydroxamates, cyclic peptides, aliphatic acids, and benzamides [13].

**Figure 1:**
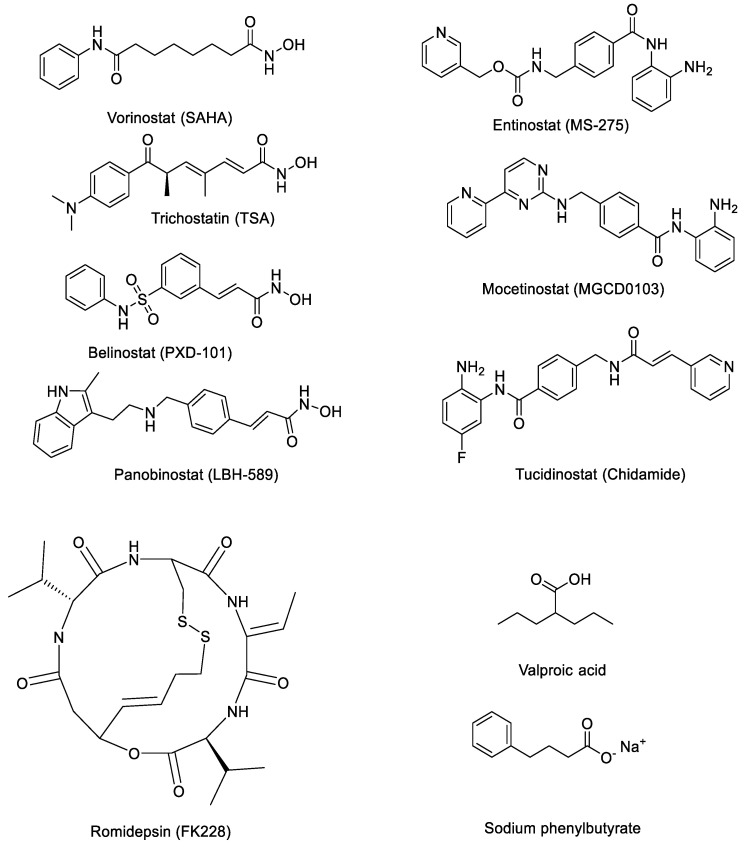
Examples of structures of major classes of HDAC inhibitors.

In Figure 1, some examples for each class are reported.

Suberoylanilide hydroxamic acid (SAHA, vorinostat), trichostatin A (TSA), and belinostat (PXD-101) are hydroxamic acid-based pan-HDAC inhibitors. Romidepsin (depsipeptide, FK228) is a natural cyclic peptide which inhibits HDAC1 and HDAC2 selectively. Entinostat, mocetinostat, and tucidinostat are benzamide derivatives. Entinostat is selective to HDAC 1, 2, and 3, and mocetinostat is a class I selective HDAC inhibitor. Aliphatic acids, including valproic acid and sodium phenylbutyrate have limited HDAC inhibitory potencies in millimolar range [14]. Some drugs such as vorinostat, romidepsin, belinostat, panobinostat, and chidamide that have been granted by US/Chinese FDA and others are under clinical trials [15].

To date, several HDAC inhibitors (HDACs) were addressed for cancer treatment, and all FDA-approved HDAC-targeting drugs are pan-HDAC inhibitors [16]. For this reason, many recent studies have focused on innovative strategies for the design of novel selective HDACIs and on their applications [17,18,19].

**Figure 2:**
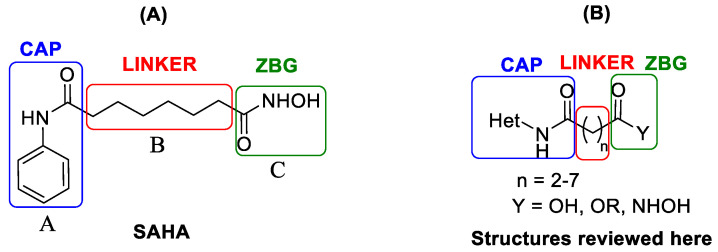
(**A**) Structure of SAHA with indication of the specific CAP-LINKER-ZBG framework. (**B**) Structure of SAHA analogos as HDACIs reported in this review.

Since most of the known HDAC isoforms show a highly conserved nature, and they bind to the main pocket of the catalytic site interacting with a Zn^2+^ ion, the classical pharmacophore model now widely accepted, shown in Figure 2 (applied to SAHA), and this reflective binding model was firstly proposed for HDAC inhibitors by Jung et al. [20,21]. The model includes three (A-B-C) key pharmacophoric features: the zinc binding group (ZBG) coordinating the catalytic zinc ion, a hydrophobic linker placed in the hydrophobic substrate binding tunnel, and a linker group connected with a CAP group occupying the entrance to the active site [17,18].

Several HDACIs contain an amide-alkyl-hydroxamic acid framework, such as that present in the first discovered HDAC inhibitor trichostatin A (TSA) (Figure 1). In this context, a very important HDACI is suberoylanilide hydroxamic acid (SAHA), well known as vorinostat, with a structure that conforms to the above indicated pharmacophore A-B-C, where A is the cap group (CAP) for protein surface interactions, C is a zinc coordinating group (ZBG) that repress the hydrolysis of acetyl group in the lysine residue, and B is a linker group that connects CAP with ZBG (Figure 2A) [22].

SAHA was the first HDAC inhibitor approved by the US Food and Drug Administration in 2006 for the treatment of cutaneous T cell lymphoma [23]. Many SAHA analogs have been synthesized and tested as HDACIs. 

The present review reports the synthesis and biological activity of HDACIs analogs of vorinostat, focusing attention on those bearing an aza-heteroaromatic instead of a phenyl group in a CAP fragment, a linear aliphatic chain of a different length as a linker, and a carboxy-, ester-, or hydroxamic group as ZBG (Figure 2B). This review, excluding patent literature, covers literature articles of the last 25 years.

We divided the review into sub-headings, depending on the length chain of the aliphatic linker. In turn, each sub-heading has been structured based on the class of the heterocycle bound to the amide nitrogen atom of the CAP group.

## 2. Two-Carbon Linker Chain

### 2-Amino-1,3,4-thiadiazoles in the CAP Group

**Scheme 1:**
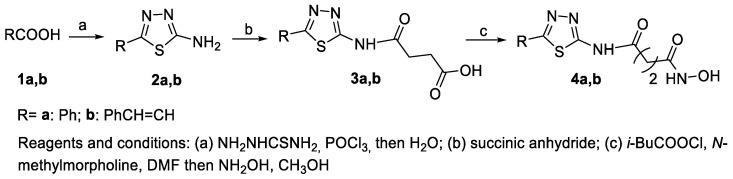
Synthetic pathway to 1,3,4-thiadiazole derivatives **4a**,**b**.

The only reported HDACIs bearing a C-2 alkyl chain are 2-amino-1,3,4-thiadiazole-based hydroxamates **4a** and **4b** [24]. They were synthesized as depicted in Scheme 1.

HDAC inhibitory activity of compounds **4a** and **4b** was assessed by the Color de Lys assay and the results showed in both cases IC_50_ values > 5 μM, lower than that of SAHA (IC_50_ = 0.15 ± 0.02). 

## 3. Three-Carbon Linker Chain

### 3.1. 2-Amino-1,3,4-thiadiazoles in the CAP GROUP

**Scheme 2:**
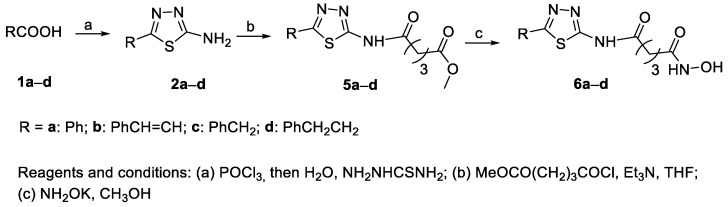
Synthetic route for preparation of compounds **6a**–**d**.

Hydroxamates bearing 2-amino-1,3,4-thiadiazole-derivatives in the CAP group and a C-3 linker chain were obtained as depicted in Scheme 2. Intermediates **5a**–**d** were obtained by the reaction between 2-amino-1,3,4-thiadiazoles **2a**–**d** and methyl 5-chloro-5-oxopentanoate (in turn obtained from dimethyl glutarate after partial hydrolysis and treatment with SOCl_2_). Treatment of **5a**–**d** with NH_2_OK in methanol gave compounds **6a**–**d** [24]. The HDAC inhibitory activity of compounds **6a**–**d** is summarized in Table 1.

Among **6a**–**d**, only **6b** showed HDAC inhibition activity (IC_50_ = 0.16 ± 0.03) close to that of SAHA (IC_50_ = 0.15 ± 0.02). Moreover, the effect of **6b** on the cell viability in MDA-MB-231 breast cancer cells and K562 chronic myelogenous leukemia cells was evaluated, resulting in a IC_50_ value of 5.90 ± 2.75 and 6.75 ± 2.37, respectively. 

### 3.2. Indazoles in the CAP Group

**Scheme 3:**
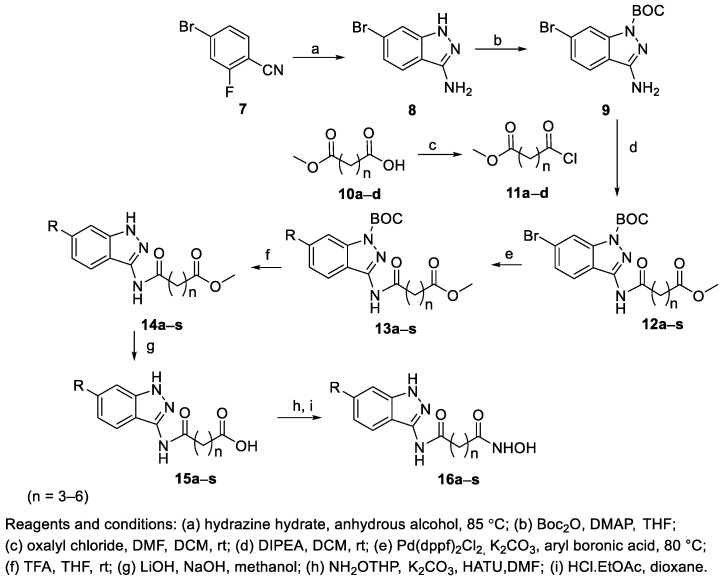
Preparation of indazole-based series **16a**–**s**.

Among the series of indazole derivatives **16a**–**s** is characterized by different spacer length and substituents on the heterocyclic ring, prepared as shown in Scheme 3; the only reported compounds with a 3-carbon linker chain are **16a** (*n* = 3, R = 3-methoxyphenyl) and related precursors [25]. 

The biological activity, of HDACIs, of compound **16a** was tested against HDAC1, HDAC2, and HDAC8 (IC_50_ = 76 nM, 168 nM, and 54 nM, respectively) and compared with that of SAHA (IC_50_ = 13 nM, 70 nM, and 44 nM, respectively). Moreover, **16a** was administered to solid cancer cell lines HCT116 (human colorectal cancer cells, IC_50_ > 50 μM), MCF-7 (human breast cancer cells, IC_50_ > 41.5 mM), and HeLa (human cervical cancer cells, IC_50_ > 50 μM), but its activity was lower than that of SAHA (IC_50_ = 4.9 mM, 0.8 mM, and 5.0 μM for the three cell lines, respectively).

## 4. Four-Carbon Linker Chain (4-C Spacer)

### 4.1. 2-Amino-1,3,4-thiadiazoles in the CAP Group

**Figure 3:**
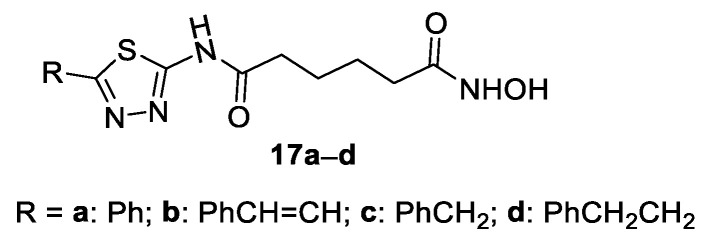
Thiadiazole hydroxamic acids with a four-methylene chain.

Compounds **17a**–**d** (Figure 3) were obtained with the same synthetic sequence shown in Scheme 2, using, in this case, 6-chloro-6-oxohexanoic acid as acyl chloride [24]. 

The HDAC inhibitory activity of the compounds was assayed: in all four cases, it was lower compared with that of SAHA which was chosen as a positive control, as indicated in Table 2.

### 4.2. Indazoles in the CAP Group

**Figure 4:**
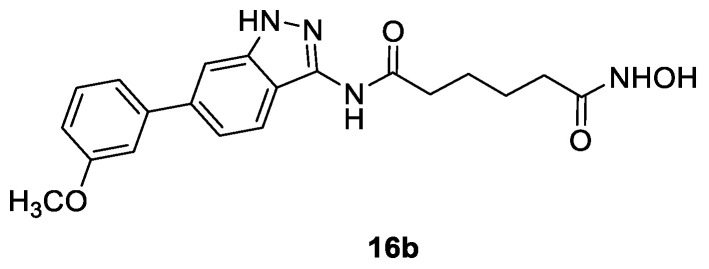
Indazole derivative **16b**.

With the synthetic sequence depicted in Scheme 3, when *n* = 4 and R = 3-methoxyphenyl, indazole derivative **16b** (Figure 4) was obtained [25]. This compound showed activity towards HDAC1 (IC_50_ = 13 nM), HDAC2 (IC_50_ = 62 nM), and HDAC8 (IC_50_ = 41 nM) equal or a little better than that of SAHA (HDAC1 IC_50_ = 13 nM; HDAC2 IC_50_ = 70 nM; HDAC8 IC_50_ = 44 nM).

### 4.3. Benzothiazoles in the CAP Group

**Scheme 4:**
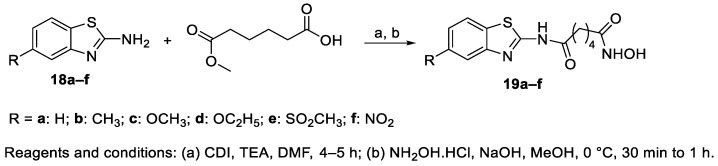
Synthesis of benzothiazolyl analogs of SAHA with the C-4 methylene linker.

Benzothiazole derivatives **19a**–**f** [26] were obtained in good yields (from 70 to 90%) by a reaction between 5-substituted 2-aminobenzothiazoles and adipic acid monomethyl ester in the presence of 1,1’-carbonyldiimidazole and triethylamine in THF followed by a conversion of the ester intermediates to the corresponding hydroxamic acids (Scheme 4).

Cytotoxicity assays of compounds **19a**–**f** against five cancer cell lines, namely SW620, MCF-7, PC3, AsPC-1, and NCI-H460 revealed that compounds **19a**–**d** exhibited cytotoxicity against all tested cancer cell lines with IC_50_ values from 7.90 to 15.12 μg/mL whereas compounds **19e** and **19f** were not cytotoxic (IC_50_ of >30 μg/mL).

However, when the effect of **19a**–**f** on histone acetylation in SW620 cells was examined, the HDAC inhibition at a concentration of 1 μg/mL was not significant.

### 4.4. 4-Anilinothieno [2,3-d]pyrimidine Derivatives in the CAP Group 

**Scheme 5:**
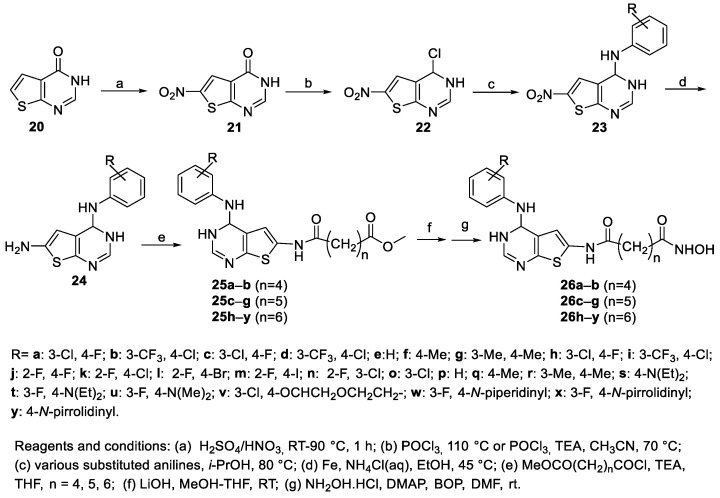
Synthetic approach to thieno [2,3-d]pyrimidine-based HDAC inhibitors with different lengths of the spacer.

Thieno [2,3-d]pyrimidine-based HDAC inhibitors with different lengths of the spacer (*n* = 2, 3, 4) [27] were synthesized from the thieno [3,2-d]pyrimidin-4(3*H*)-one **20** after nitration in an alpha position to the thiophene ring, chlorination on the pyrimidine ring, treatment with different anilines, and reduction in the nitro group to the amino group. The latter was reacted with the acyl chloride MeOCO(CH_2_)_n_COCl (*n* = 4–6) to form the methyl ester intermediates **25** that were subjected to hydrolysis. Subsequent treatment of the obtained acid with hydroxylamine hydrochloride in the presence of BOP and DMAP afforded the targeted compounds **26** (Scheme 5).

Focusing attention on the C-4 spacer, the in vitro inhibitory activity of compounds **26a** (R = 3-Cl, 4-F) and **26b** (R = 3-CF_3_, 4-Cl) against HDAC1, HDAC3, and HDAC6 was lower with respect to that of SAHA, except for **26a** towards HDAC3 (**26a** IC_50_ = 126.56 ± 9.04; SAHA IC_50_ = 158.17 ± 6.66).

### 4.5. Thiazolyl-Coumarin Derivatives in the CAP Group

The in vitro inhibitory activity against HDACs of thiazolyl-coumarins linked, through a C-4 alkyl spacer, to classic zinc binding groups, such as hydroxamic and carboxylic acid moieties, was evaluated [28].

**Scheme 6:**
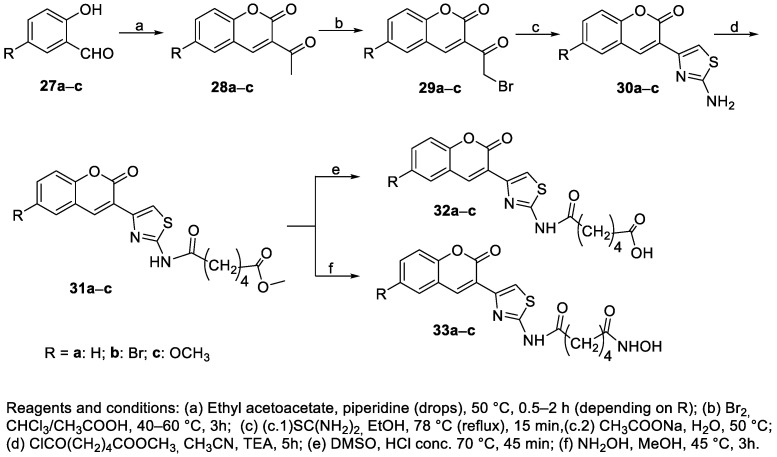
Synthetic route to thiazolyl derivatives **32** and **33**.

In particular, compounds **32a**–**c** and **33a**–**c** were synthesized as shown in Scheme 6. 

The first step was a Knoevenagel-type condensation between salicyl aldehydes **27a**–**c** and ethyl acetoacetate. After bromination of the coumarin acetyl group, the Hantzsch synthesis gave the thiazole intermediates **30a**–**c** that was reacted with the acyl chloride derived from adipic acid methyl ester to give **31a**–**c**. From the latter, acids **32a**–**c** and hydroxamic acids **33a**–**c** were obtained. Compounds **33a**–**c** were the most active inhibitory compounds of the HDACs towards the HeLa cells.

In addition, it has been shown that the expression and the activity of distinct histone deacetylases (HDACs) are strongly correlated with cardiac fibrosis (CF) development. In particular, HDAC1 and HDAC2 are mainly associated with the regulation of the biology of CF in the heart; in this context, compound **33a** showed significant inhibition on CF proliferation at 1 µM concentration and also a decrease in procollagen type I and α–smooth muscle actin (α–SMA) expression levels.

## 5. Five-Carbon Linker Chain (5-C Spacer)

### 5.1. 2-Amino-1,3,4-thiadiazoles in the CAP Group

**Figure 5:**
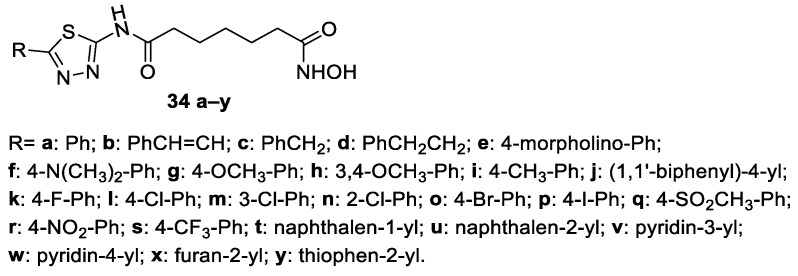
2-Amino-1,3,4-thiadiazoles connected to a C-5 spacer through an amide bond.

Compounds **34a**–**d** (Figure 5) were obtained as shown in Scheme 2, using, in this case, 7-chloro-7-oxohexanoic acid as acyl chloride [24]. The relevant HDAC inhibitory activity is reported in Table 3.

The HDAC inhibitory activity evaluation of compound **34a** (R = Ph, IC_50_ = 0.089 ± 0.005 μM) achieved a better result than that of SAHA (IC_50_ = 0.15 ± 0.02 μM), similarly to in cases **34c** and **34d**; in contrast, **34b** was >5 μM. 

Other compounds bearing 1,3,4-thiadiazole ring as the surface recognition motif, obtained with a sequence very similar to that depicted in Scheme 2, were compounds **34e**–**y** (Figure 5) [29].

Among them, only **34g** (R = 4-OCH_3_), **34i** (R = 4-CH_3_), **34v** (R = pyridin-3-yl), **34w** (R = pyridin-4-yl), **34x** (furan-2-yl), and **34y** (thiophen-2.yl) showed IC_50_ values IC_50_ of HDACs close to that of SAHA. 

### 5.2. 4-Anilinothieno [2,3-d]pyrimidine Derivatives in the CAP Group

The inhibitory activity of compounds **26c**–**g**, synthesized as depicted in Scheme 5, was tested for HDAC1, HDAC3, and HDAC6. In all cases, it was higher than that of SAHA, as reported in Table 4 [27].

### 5.3. Indazole Nucleus in the CAP Group

**Figure 6:**
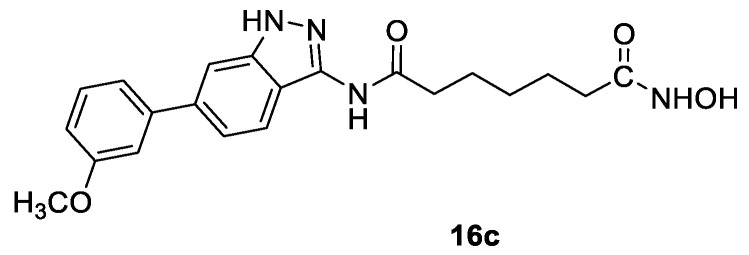
Indazolyl SAHA analog **16c**.

Indazolyl compound **16c** (Figure 6) was obtained through the synthetic sequence depicted in Scheme 3 when *n* = 5 and R = 3-methoxyphenyl [25].

This compound showed activity towards HDAC1 (IC_50_ = 2.6 nM), HDAC2 (IC_50_ = 6.3 nM), and HDAC8 (IC_50_ = 4.5 nM) that was higher than that observed for the corresponding homologues **16a** and **16b**, thus indicating a strong effect of the chain length in inducing biological activity.

## 6. Six-Carbon Linker Chain (6-C Spacer)

### 6.1. 2-Amino-1,3,4-thiadiazoles in the CAP Group

**Figure 7:**
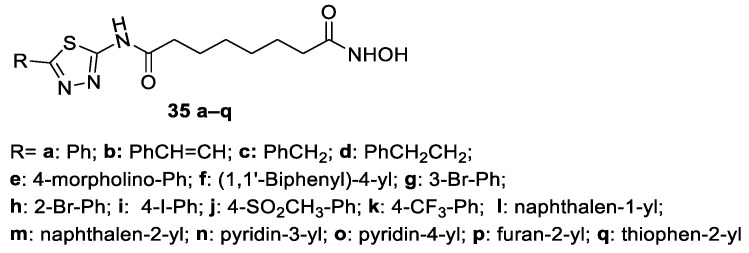
2-amino-1,3,4-thiadiazoles connected to a C-6 spacer through an amide bond, with hydroxamic acid as the ZBG group.

Compounds **35a**–**d** (Figure 7) were obtained as shown in Scheme 2, using, in this case, 8-chloro-8-oxohexanoic acid as acyl chloride [24], and the relevant data on their HDAC inhibitory activity are summarized in Table 5. Compounds **35 e**–**q** were obtained in a similar manner [29].

Moreover, among compounds **35e**–**q**, only **35n** (R = pyridin-3-yl), **35o** (R = pyridin-4-yl), and **35q** (R = thiophen-2-yl) showed an IC_50_ (referred to HDACs) value lower that that found for SAHA. This recalls the behavior found for **34v**, **34w**, and **34y**, bearing the same CAP group but with a 5-C spacer, indicating the efficacy of pyridine and thiophene derivatives. The viability of cancer cells MDA-MB-231, K562, and PC3 was measured by MTT assay for compounds **34v**, **34w**, **34y**, **35n**, **35o**, and **35q**. The results showed that **35q** had higher efficacy than SAHA on all cell lines tested, whereas **35o** only had higher efficacy on MDA-MB-231 cells.

**Scheme 7:**
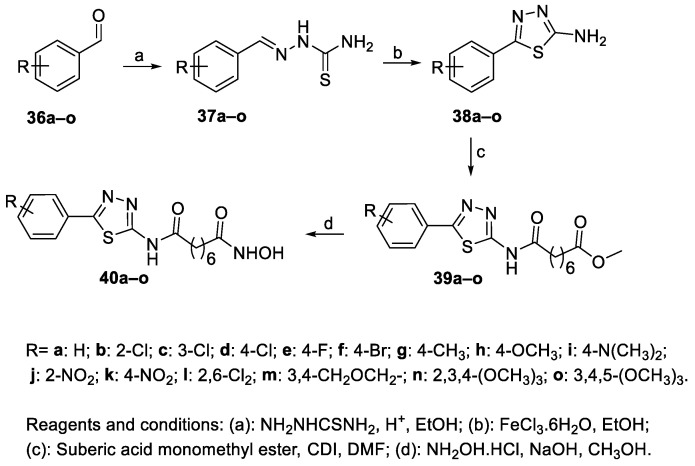
Synthetic pathway for N1-hydroxy-N8-(5-substituted phenyl-1,3,4-thiadiazol-2-yl)octandiamides **40a–o**.

Thiadiazole derivatives **40a**–**o** were synthesized as depicted in Scheme 7 starting from benzaldehyde **36a** (or differently substituted benzaldehydes **36b**–**o**) and thiosemicarbazide followed by cyclization to thiadiazole derivatives **38a**–**o**. The latter were reacted with 1,10-carbodiimidazole (CDI) and suberic monomethyl ester acid to obtain derivatives **39a**–**o**, from whose final hydroxamates **40a**–**o** were obtained [30].

In this series, compounds **40b**, **40c**, and **40d** were found to possess potent anticancer cytotoxicity and HDAC inhibition effects. They were generally two- to five-fold more potent in terms of cytotoxicity compared to SAHA against five cancer cell lines tested (SW620, colon cancer; MCF-7, breast cancer; PC3, prostate cancer; AsPC-1, pancreatic cancer; and NCI-H460, lung cancer). Docking studies revealed that these hydroxamic acids displayed higher affinities than SAHA towards HDAC8.

### 6.2. Thiazoles in the CAP Group

**Scheme 8:**
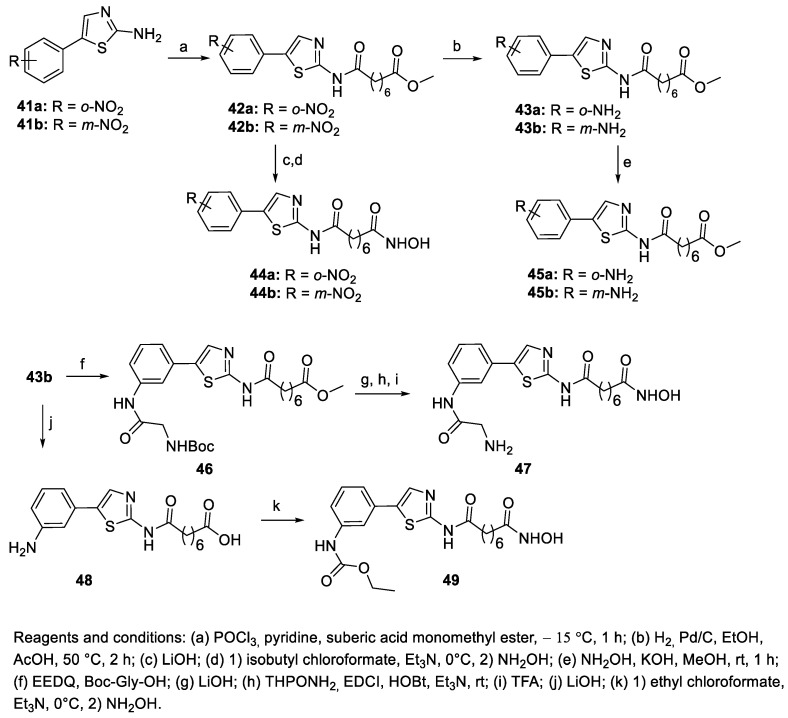
Synthetic sequence for preparation of phenylthiazole derivatives.

Phenylthiazole-bearing hydroxamates [31] *Ortho*- and *meta*-amino-substituted phenylthiazole derivatives **41**–**49** were synthesized starting from commercial 4-(2-nitrophenyl)thiazol-2-ylamine and 4-(3-nitrophenyl)thiazol-2-ylamine (Scheme 8).

**Scheme 9:**
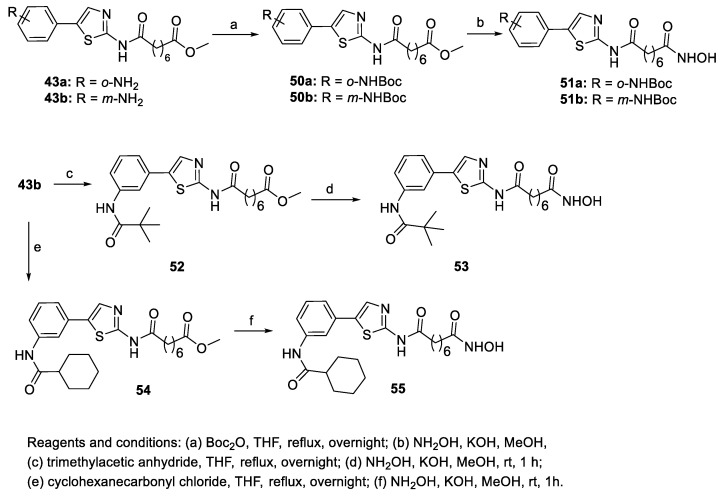
Synthesis of compounds **50**–**55**.

In the same paper, phenylthiazoles **50**–**55** bearing an amide or urethane residue on the benzene ring in linkage with a bulkier alkyl group have been reported. They were synthesized starting from compounds **43a** and **43b** through the sequence shown in Scheme 9.

The inhibitory activity of the above compounds has been tested towards HDAC1, HDAC2, HDAC3, HDAC8, HDAC10, and HDAC6. In comparison with the unsubstituted phenylthiazole analog, the introduction of an amino group as in **45a** and **45b** or a glycineamide residue as in **47** did not produce significant changes in both activity and isoform selectivity. The *ortho*-nitro compound **44a** is almost 10-fold less potent than the corresponding amine analog **45a**. The meta-substituted ethyl carbamate **49** showed an activity against HDAC1 and HDAC2 very close to that of its amine analog **45b**, but it showed a 3-fold improvement in its HDAC6 inhibitory activity. Ongoing from the ethyl- (**49**) to the *tert*-butyl- (**50b**) carbamate, an increase in HDAC6 inhibitory activity was observed, but there were no changes in inhibitory activity towards HDAC1 and HDAC2. Moreover, the introduction of a Boc-protecting group led to an enhancement in the inhibitory activity towards HDAC6 (>15-fold in **51b** in comparison with **45b**). Interestingly, replacement of the *tert*-butyloxy group of **51b** by a cyclohexyl group as in **55** leads to subnanomolar potency against both HDAC2 and HDAC3 (IC_50_ values 200-fold increase against HDAC2 and >20-fold increase against HDAC3), while the IC_50_ value for HDAC6 was still below 0.2 nM. Compound **51a** showed a 2-fold decrease in activity towards HDAC1 and HDAC2, with similar inhibitory potency against HDAC6 relative to the unprotected *ortho*-NH_2_ ligand **45a**. Also, conversion of **51b** to the pivaloyl derivative **53** produced a >10-fold decrease in HDAC6 inhibition. Inhibitory data of compound **44b** have been also reported in a previous study [32].

Compounds **44a**, **45a**, **45b**, and **49** have been also tested towards five pancreatic cancer cell lines, and their antiproliferative activity was compared with that of SAHA and showed similar or improved potencies relative to SAHA. Among them, the *meta*-amino-substituted phenylthiazole **45b** gave the best IC_50_ value against the Mia Paca-2 cell line (IC_50_ = 10 nM), while its carbamate analog **49** showed the best overall inhibitory activity against all five pancreatic cancer cell lines.

**Scheme 10:**
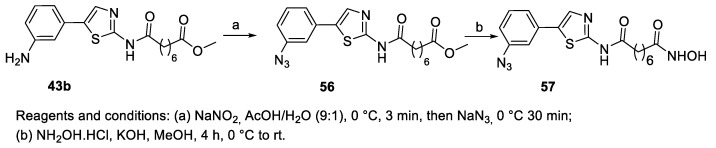
Synthesis of an azide-containing compound **57**.

In another study [33], the phenylthiazole-based probe **57**, with an azide group on the phenyl ring, was designed to mimic the scaffold of SAHA. The synthesis is shown in Scheme 10. 

Compound **57** resulted in 6.1-fold less activity in inhibiting HDAC8 compared to SAHA, and this behavior has been attributed to an increase in the lipophilic nature of the solvent exposed surface binding group that influences to the overall binding affinity.

Finally, hydroxamic derivatives bearing both unsubstituted- and *p*-*N*-pyrrolidinyl-substituted phenylthiazole amide functionality showed better HDAC potency and cellular activity (towards HT1080 and MDA435 cells) with respect to SAHA [34].

### 6.3. Pyrazole Nucleus in the CAP Group

HDACIs bearing pyrazole and isoxazole derivatives in the CAP group have been synthesized and studied by Petukhov et al. [35,36]. 

**Scheme 11:**
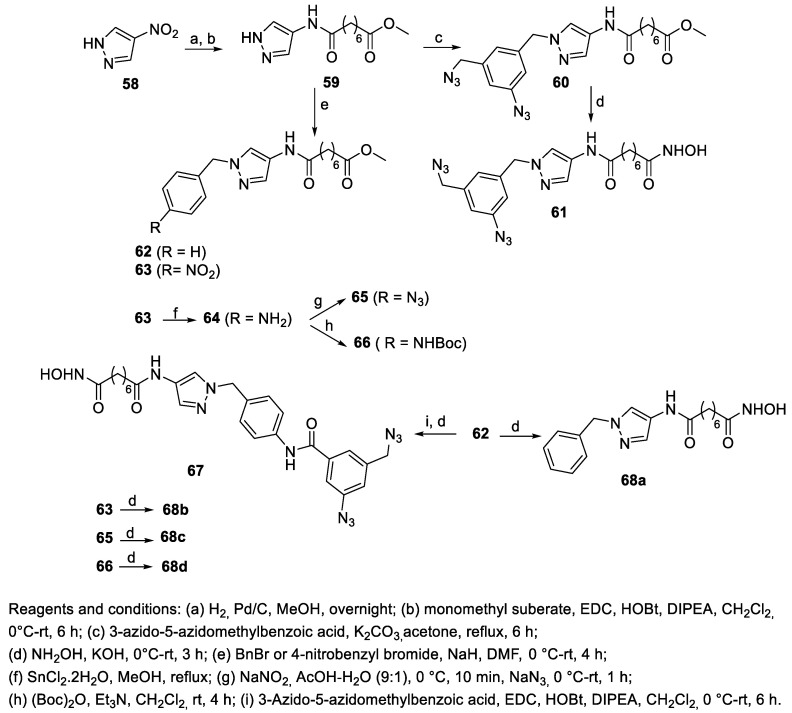
Multistep route to SAHA analogs **61, 67**, and **68a**–**d** with a pyrazole nucleus in the CAP group.

In particular, compounds with pyrazole nucleuses **61, 67,** and **68a**–**d** have been synthesized according to Scheme 11 starting from commercially available 4-nitropyrazole (**58**). The synthetic strategy involves, as the first step, an amide coupling between 4-aminopyrazole, obtained from **58** through hydrogenolysis, and monomethyl suberate to give (**59**). Alkylation of **59** with toluene-4-sulfonic acid 3-azido-5-azidomethylbenzyl ester in the presence of K_2_CO_3_ obtained compound **60**. Compounds **62** and **63** were obtained by alkylation of **59** with benzyl bromide or 4-nitrobenzyl bromide in the presence of NaH in DMF, respectively. A reduction in the nitro group of **63** gave aniline **64**, a key intermediate for compounds **65** and **66**. Diazotization of the amino group of the aniline derivative **64** followed by an azide displacement reaction with NaN_3_ gave the corresponding azido compound **65**. Treatment of **64** with Boc anhydride furnished the carbamate **66**. Compound **67** was obtained by an amide coupling between **64** and 3-azido-5-azidomethylbenzoic acid followed by treatment with KOH/NH_2_OH in MeOH. The same treatment on the methyl esters **60**, **62**, **63**, **65**, and **66** gave the corresponding hydroxamates **61** and **68a-d**, respectively.

Pyrazoles **61**, **67**, and **68a**–**d** were tested for the inhibition of HDAC3 and HDAC8 isoforms. The inhibition of HDAC8 was measured using the fluorescent acetylated HDAC substrate Fluor de Lys and the commercially available recombinant human HDAC8, whereas the inhibition of HDAC3 was measured using the fluorescent HDAC substrate Boc-L-Lys(Ac)-AMC and the commercial recombinant human HDAC3/NCoR2.

The results are summarized in Table 6. 

The simplest benzyl substituted pyrazole **68a** inhibited HDAC3 and HDAC8 with IC_50s_ of 44 and 76 nM, respectively. Introduction of a nitro group at the 4-position of the benzyl group of **68a** gave compound **68b** that showed slightly lower activity for both isoforms, whereas the corresponding azido compound **68c** exhibited a 2.0- and 2.7-fold better potency, with its IC_50_ values being 22 and 28 nM for HDAC3 and HDAC8, respectively. Overall, compounds **68a**–**c** exhibited an inhibitory activity against HDAC3 comparable to that of SAHA but exhibited a better double digit nanomolar activity against HDAC8. Introduction of a bulky Boc-protected amino group in **68d** decreased the HDAC activity by about 10-fold. Replacement of the Boc group with a lipophilic aromatic diazide as in **67** further decreased the activity for both HDAC3 and HDAC8 to 432 and 487 nM, respectively. Comparison of the activity data of **68b**,**c** with **68d** and **67** shows that the presence of the bulky substituent in the *para* position of the terminal phenyl ring leads to the lower activities for both HDAC3 and HDAC8 isoforms. The replacement of the phenyl group with a 3-azido-5-azidomethyl phenyl group, resulting in **61**, revealed that this compound was 8-fold more active towards HDAC8 than for HDAC3, with IC_50s_ equal to 17 and 128 nM, respectively. The activity of the methyl ester **60** towards HDAC8 was 36.0 ± 2.20 μM [37].

Compound **61**, also called SAHA diazide, was also tested against HDAC1 and HDAC4; compared with the activity of SAHA (*K*i = 0.051 and >30 μM for HDAC1 and HDAC4, respectively), *K*i values for **61** were *K*i = 0.14 and 13.05 μM for HDAC1 and HDAC4, respectively. 

### 6.4. Pyridine and Pyrimidine Nucleus in the CAP Group

**Figure 8:**
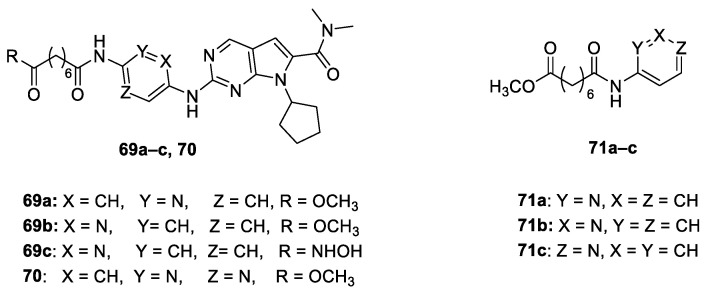
Pyridine derivatives tested against HDAC1.

The synthesis and biological activity of compounds **69a**–**c**, bearing a pyridinyl substituent in the CAP group (Figure 8), have been reported, but their activity towards HDAC1 was much lower than that of SAHA [38].

Similar behavior was also found for the pyrimidine derivative **70**. 

The simpler pyridinyl derivatives **71a**–**c** (Figure 8) were profiled using a partially purified HDAC enzyme obtained from H1299 cell lysate in antiproliferative assays (towards H1299 and HCT116) and in a p21 promoter induction assay [39].

In these cases, the activity towards enzyme was comparable to that of SAHA. The 2-pyridyl isomer **71a** was essentially equipotent to SAHA in the promoter assay, but 3-fold less potent in HCT116 growth inhibition and >10 μM in H1299 growth inhibition. The 3- and 4-pyridyl isomers **71b** and **71c** were less potent than SAHA. The difference in cellular activity of these positional isomers has been hypothesized due to differences in cellular permeability or intracellular metabolism of the compounds.

### 6.5. Thienopyrimidine Nucleus in the CAP Group

The biological activity of thienopyrimidine derivatives **26h**–**y**, synthesized as reported in Ref. [18] and depicted in the above Scheme 5, have been tested as inhibitors of HDAC1, HDAC3, and HDAC6, and of proliferation of RMPI8226 and HCT-116 cancer cells. In all cases, the activity found was comparable with that of SAHA.

**Figure 9:**
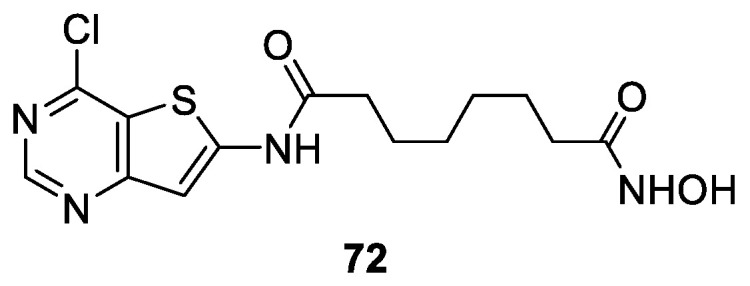
Structure of compound **72**.

In the same paper, the biological activity of compound **72** (Figure 9) was also tested and the results showed poor inhibitory activity in many cases, suggesting that the presence of the 4-aniline fragment could increase the lipophilic interaction with HDACs to induce good inhibitory activities against them.

**Scheme 12:**
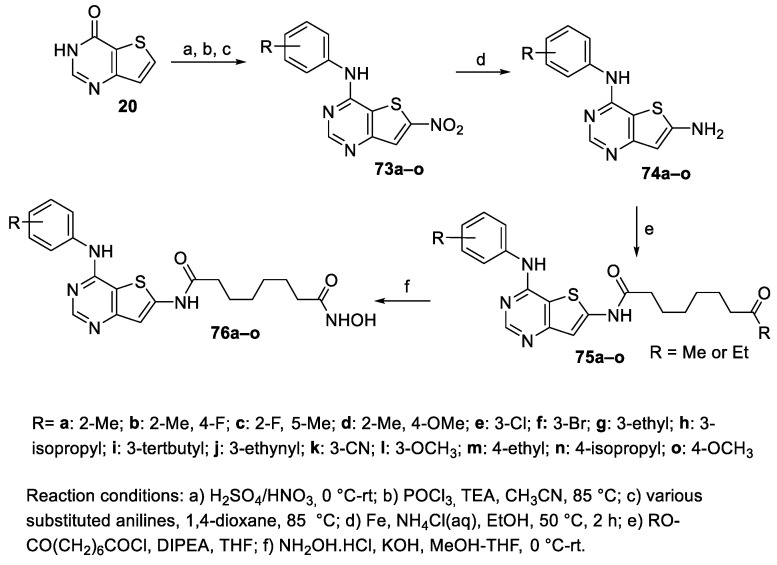
SAHA analogs with thienopyrimidine nucleus in CAP group.

The above cited paper was followed by a second [40], only focused on the C-6 spacer, in which the fifteen novel compounds **76a**–**o**, bearing the thienopyrimidine fragment on the CAP group were synthesized from methyl 3-aminothiophene-2-carboxylate that, after cyclization with formamidine acetate under microwave conditions, gave the thieno [3,2-d]pyrimidin-4(3*H*)-one (**20)** in similar conditions to those already reported in Scheme 5. The latter was subjected to nitration and subsequent chlorination then coupled with a series of anilines to give compounds **73a**–**o**. A reduction in the nitro group to the amino group afforded the key precursors **74a**–**o**. After treatment with acyl chlorides amides, **75a**–**o** were obtained. Lastly, the target products **76a**–**o** were obtained after reaction with hydroxylamine hydrochloride (Scheme 12).

The ability of compounds **76a**–**o** to inhibit recombinant human HDAC1, HDAC3, and HDAC6 isoforms and ‘in vitro’ activity against cancer cell lines RMPI 8226 and HCT 116 was tested. Most of them displayed good inhibitory and anticancer activities, particularly compound **76j** that showed IC_50_ values (29.81 ± 0.52 nM, 24.71 ± 1.16 nM, and 21.29 ± 0.32 nM for HDAC1, HDAC3, and HDAC6, respectively) much lower than those found for SAHA (195.00 ± 16.12 181.05 ± 28.92 105.10 ± 25.46). Moreover, the IC_50_ values of compound **76j** against RPMI 8226 and HCT 116 proliferation were 0.97 ± 0.072 mM and 1.01 ± 0.033 mM, respectively, and it up-regulated the level of histone H3 acetylation at the concentration of 0.3 mM.

### 6.6. Indazole Nucleus in the CAP Group

**Figure 10:**
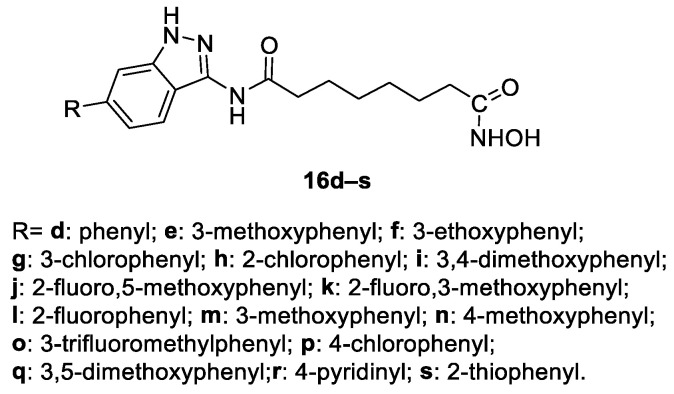
Indazolyl SAHA analogs **16d**–**s**.

In Figure 10, indazolyl derivatives **16d**–**s** are shown, synthesized through the approach depicted above in Scheme 3 [25].

Among compounds **16d**–**s**, compounds **16n** and **16p** emerged as excellent inhibitors of HDAC1 (IC_50_ = 2.7 nM and IC_50_ = 3.1 nM), HDAC2 (IC_50_ = 4.2 nM and IC_50_ = 3.6 nM), and HDAC8 (IC_50_ = 3.6 nM and IC_50_ = 3.3 nM). Antiproliferation assays revealed that these compounds also showed antiproliferative activities against HCT-116 and HeLa cells better than SAHA. Moreover, compounds **16n** and **16p** up-regulated the level of acetylated α-tubulin and histone H3 and promoted cell apoptosis.

**Figure 11:**
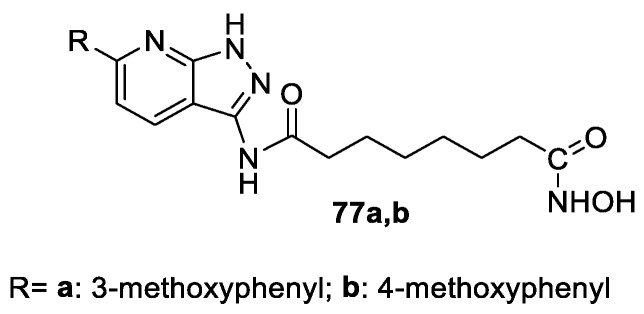
Structure of compounds **77a,b**.

According to a similar synthetic route similar to that of Scheme 3, 1*H*-pyrazolo [3,4-b] pyridine derivatives **77a**,**b** (Figure 11), bioisosters of compounds **16e** and **16n**, respectively, were obtained from 2,6-dichloronicotinonitrile through a multistep sequence [25].

The inhibitory activities of **77a** and **77b** towards HDACs slightly decreased, indicating that the presence of the 6-phenyl-1*H*-indazole scaffold is important to affecting the biological activity.

### 6.7. Benzothiazole Moiety in the CAP Group

**Figure 12:**
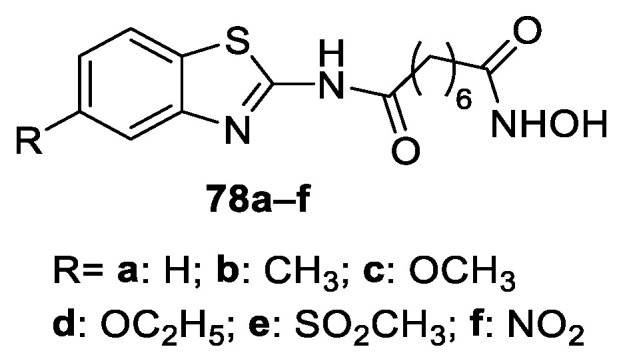
SAHA analogs with benzothiazolyl scaffold in CAP group.

Compounds **78a**–**f** (Figure 12) were obtained from 2-aminobenzothiazole derivatives with the sequence depicted in Scheme 4, with the difference to use suberic acid monomethyl ester instead of adipic acid monomethyl ester [26]. 

It was observed that several compounds showed good inhibition against HDAC3 and HDAC4. The amount of enhanced acetylation of histone-H3 and -H4 in SW620 cells by **78a**–**c** and **78f** was similar to that found for SAHA.

Moreover, all six compounds displayed cytotoxicity against five cancer cell lines (SW620, colon cancer; MCF-7, breast cancer; PC3, prostate cancer; AsPC-1, pancreatic cancer; NCI-H460, lung cancer), with average IC_50_ values ranging from 0.59 to 11.08 μg/mL. 

Homologues 4C-bridged compounds showed slight or no increase in histone acetylation, suggesting that the linker length between the benzothiazol and hydroxamic moieties required for good HDAC inhibition of this compound series was similar to that of SAHA. In addition, the size of the 6-substituents on the benzene ring rather than their electronic effects was important for HDAC binding; for example, **78d** and **78e** bearing relatively larger substituents (–OC_2_H_5_ and –SO_2_CH_3_) compared to the other compounds in the series did not inhibit HDAC activity. Actually, compounds **78c** (bearing –OCH_3_, an electron-donating group) and **78f** (bearing –NO_2_, an electron-withdrawing group) showed similar HDAC inhibitor power and were almost equally cytotoxic.

### 6.8. Benzoxazole Moiety in the CAP Group

**Scheme 13:**
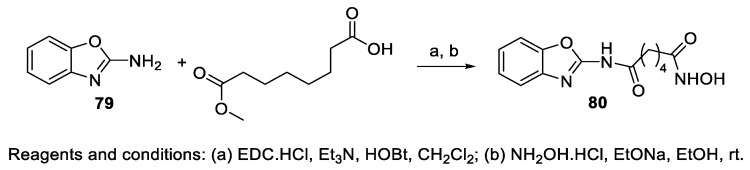
Benzoxazole analog of SAHA from 2-aminobenzoxazole.

From the reaction between 2-aminobenzoxazole and suberic acid monomethyl ester and the subsequent transformation of the methyl ester to hydroxamic group, compound **80**, which can be considered a bioisoster of **78a**, was obtained (Scheme 13) [41]. 

Compound **80** was an inhibitor of human HDAC1; HDAC2 more potent than vorinostat and was also comparable as an inhibitor of HDAC6. It was a slightly more potent inhibitor than vorinostat on the growth of A549, Caco-2, and SF268 cells and was chosen for further studies against two colon cancer cell lines, HCT116 GNAS R201C/+ and LS174T cells, that genetically resemble PMP tumor cells, and it proved to be a more potent antiproliferative compound than vorinostat in both cases.

### 6.9. Isoquinoline Moiety in the CAP Group

**Scheme 14:**
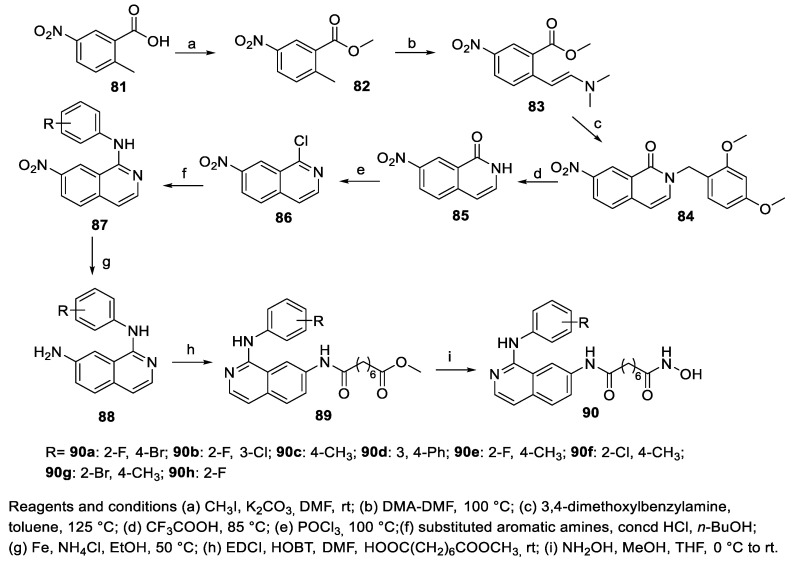
Synthetic way to SAHA analogs **89** and **90** with isoquinoline scaffold in the CAP group.

Novel HDACIs bearing isoquinoline fragments in CAP groups have been synthesized starting from 2-methyl-5-nitrobenzoic acid (**81**) [42]. After esterification to **82** followed by treatment with DMA-DMF and cyclization with 3,4-dimethoxylbenzylamine, intermediate **84** was obtained. The latter was deprotected to **85** then chlorinated to **86**, which was coupled with a series of anilines to generate compounds **87**. A reduction in the nitro group followed by reaction with 8-methoxy-8-oxooctanoic acid afforded the amides **89** which, after treatment with freshly prepared hydroxylamine, gave compounds **90a**–**h**. (Scheme 14) [42].

Compounds **90a**–**h** were tested against HDAC1, HDAC3, and HDAC6 and all showed better activity than SAHA, which was used as a positive control. The best active compound was **90c**, showing IC_50_ values 4.17 nM, 4.00 nM, and 3.77 nM against HDAC1, HDAC3, and HDAC6, respectively. Furthermore, the antiproliferative activity of compounds **90a**–**h** against multiple myeloma cell line RPMI 8226 was tested and the more active were **90a**, **90f**, and **90g** with IC_50_ values 0.46 μM, 0.52 μM, and 0.47 μM, respectively. 

**Figure 13:**
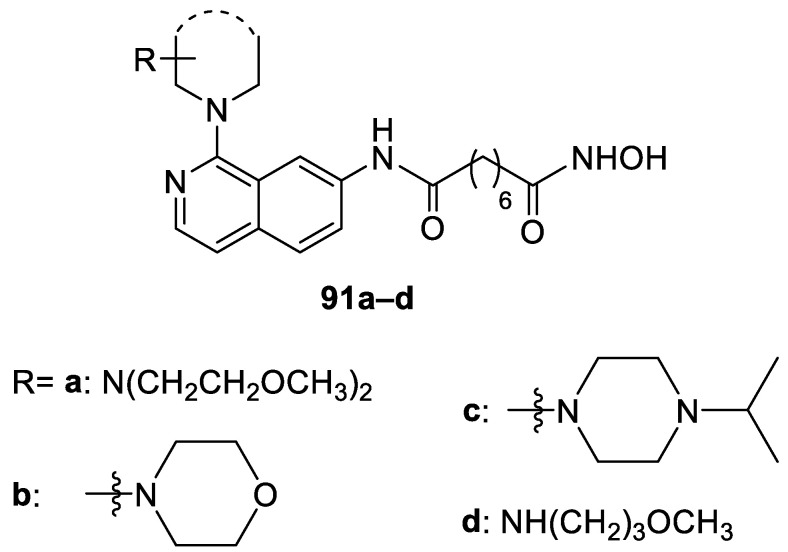
Isoquinoline derivatives **91a**–**d**.

When intermediate **86** was reacted with aliphatic amines under microwaves conditions, after a reduction in the nitro group to amino group and subsequent treatment as reported in steps h and i of Scheme 14, isoquinolines **91a**–**d** (Figure 13) were obtained.

Compound **91a** with a large substituent at the C-1 position of the isoquinoline ring significantly decreased with respect to **91b**–**d** inhibitory activities against HDACs as well as the proliferation of RPMI 8226 cells. Compounds **91b**–**d** displayed similar enzymatic activities, suggesting that small aliphatic amines at the C-1 position do not significantly affect the inhibitory activities against HDACs enzyme in vitro and the proliferation of the cancer cells with respect to compounds **90a**–**h**, bearing an aromatic substituent at the C-1 position.

**Figure 14:**
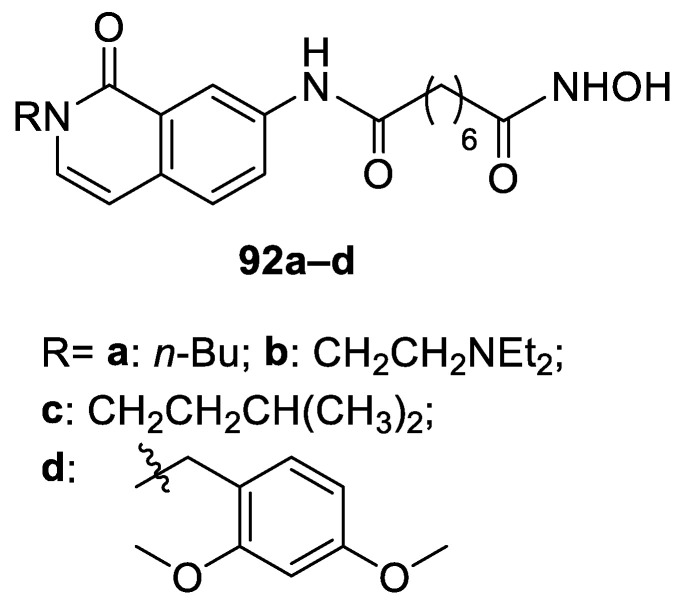
Isoquinoline-1(2H)-one derivatives **92a**–**d**.

Finally, to test the effects of the spatial orientation of the *N*-substituents, compound **83** depicted in Scheme 14 was reacted with different aliphatic amines in toluene at 110 °C and the obtained intermediate subjected to steps g, h, and i (reported Scheme 14), thus obtaining isoquinoline-1(2*H*)-one derivatives **92a**–**d** (Figure 14) 

The inhibitory activity of the series **92a**–**d** towards HDAC1, HDAC3, and HDAC6 isoforms and cancer cell proliferation were evaluated. These compounds exhibited weaker inhibitory activities against HDACs, indicating that the binding affinity between the *N*-substituent isoquinoline-1-one scaffold and the HDAC surface was decreased with respect to the **91a**–**d** series.

**Figure 15:**
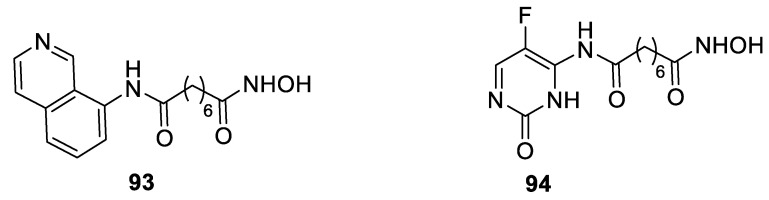
Isoquinoline and pyrimidinone derivatives **93** and **94**.

In a paper focused on the study of the influence of the substitution of the phenyl SAHA capping group with various substituents [43], two compounds bearing heterocyclic rings have been reported, one (**93**) with a isoquinolinyl group and the other (**94**) with a pyrimidin-2(1*H*)-one moiety (Figure 15), but both displayed a very weak antiproliferative and histone deacetylation activities.

### 6.10. Quinazoline Moiety in the CAP Group

**Scheme 15:**
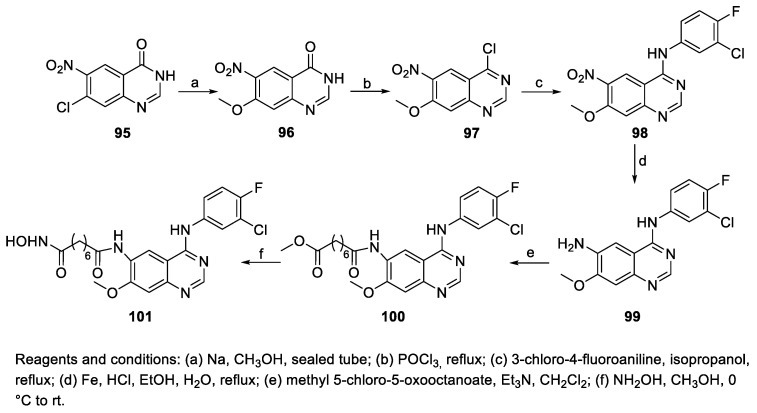
Synthetic scheme to quinazoline derivative **101**.

Taking into account the known role of hydroxamic acids as HDAC inhibitors and that of quinazolines as EGFR/HER2 inhibitors, some authors synthesized compounds bearing both functionalities in order to find efficient multitarget inhibitors [44]. Thus, among various compounds, they prepared quinazoline derivative **101** starting from **95** with the multistep procedure depicted in Scheme 15.

The HDAC inhibitory activity of the quinazoline SAHA analog **101** was determined using the Biomol Color de Lys system and the IC_50_ value was 15.3 nM. This compound also exhibited EGFR and HER2 kinase activity.

## 7. Seven-Carbon Linker Chain (7-C Spacer)

### Pyridine and Pyrimidine Moiety in the CAP Group

**Scheme 16:**
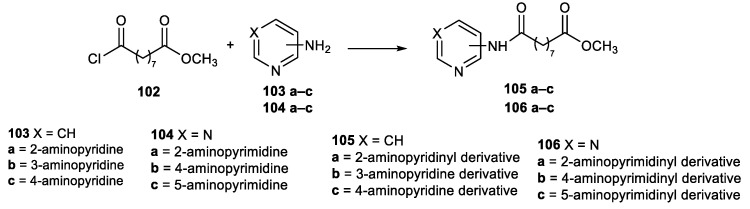
Synthesis of pyridine and pyrimidine derivatives **105** and **106**.

A series of compounds bearing pyridine or pyrimidine moiety bound to an azelayl scaffold through Schotten–Bauman-like reaction was synthesized as reported in Scheme 16 [45]. The series was subjected to biological screening on a panel of tumor cell lines: noticeably, none of the compounds induced cytotoxicity in the normal fibroblast cell line, while only osteosarcoma cells (U2OS) appeared to be sensitive to compound **106a**.

Compound **106a** was studied ‘in silico’, by using histone deacetylases as molecular target, which revealed that it is able to interact with HDAC 7, which is in agreement with studies which have disclosed an unexpected function for HDAC7 in osteoclasts. 

## 8. Conclusions

This review is focused on the synthesis and biological activity, in terms of HDAC inhibition, of SAHA analogs bearing as a linker a linear aliphatic chain of different lengths. The CAP group was selected among those in which the amide was directly bound to a heterocycle. Heterocycles present in the CAP group herein considered belonging to the classes of 1,3,4-thiadiazoles, indazoles, thiazoles, and their benzoderivatives, benzoxazoles, 4-anilinothienopyrimidines, pyrazoles, pyridines, pyrimidines, isoquinolines, and quinazolines. The ZBG is the ester, carboxylic, or hydroxamic acid group. Biological data reported in the considered literature mainly referred to hydroxamic acid derivatives, and the data were usually compared to the SAHA activity chosen as a reference. In some papers, ‘in vitro’ activity towards selected cancer cell lines was also evaluated.

In agreement with the knowledge that HDACIs often suffer from their multi-directional selectivity, in many of the cases herein, the selectivity towards a single HDAC isoform were poor. 

The influence of the aliphatic chain length of the linker is evident in compounds bearing the same cap and ZGB groups. Thus, by comparing data of Table 1, Table 2, Table 3 and Table 5, it can be deduced that compounds with the linker composed of five or six methylene units inhibited HDAC more efficiently than those characterized by C-2, C-3, and C-4 linker.

Analogous behavior was observed for compounds bearing indazoles as the CAP group, whose biological data are summarized in Table 7.

The linker between the CAP group and zinc-binding group strongly affects the inhibitory activity on HDACs: on increasing the length of the linker, the better inhibitory activity was obtained for a methylene chain with six carbon atoms. The authors think that the linker might affect the orientation of the CAP group and zinc-binding group, influencing the binding affinity between the protein and ligand. Through molecular docking and dynamic studies, the authors stated that the potent HDAC inhibitory activities are mainly caused by van der Waals and electrostatic interactions with the HDACs.

The table also reports the ‘in vitro’ biological activity against the proliferation of a panel of cancer cell lines: the behavior reflects what was observed for HDAC inhibition activity.

Because of the growing importance to develop selective HDAC inhibitors, many studies are also investigating this area, and in our introduction we reported some recent papers on this topic.

## Figures and Tables

**Table 1:** HDAC inhibitory activity of compounds **6a**–**d**.

Compound	HDAC IC_50_ (μM)
**6a**	>5
**6b**	0.16 ± 0.03
**6c**	1.87 ± 0.41
**6d**	2.71 ± 0.25
SAHA	0.15 ± 0.02

**Table 2:** HDAC inhibitory activity of compounds **17a**–**d**.

Compound	HDAC IC_50_ (μM)
**17a**	1.03 ± 0.04
**17b**	1.70 ± 0.40
**17c**	1.12 ± 0.01
**17d**	3.49 ± 0.04
SAHA	0.15 ± 0.02

**Table 3:** HDAC inhibitory activity of compounds **34a**–**d**.

Compound	HDAC IC_50_ (μM)
**34a**	0.089 ± 0.005
**34b**	>5
**34c**	0.22 ± 0.04
**34d**	0.33 ± 0.05
SAHA	0.15 ± 0.02

**Table 4:** ‘In vitro’ inhibitory activity against HDACs of compounds **26c**–**26g**.

Compound	R	HDAC1 IC_50_ (nM)	HDAC3 IC_50_ (nM)	HDAC6 IC_50_ (nM)
**26c**	3-Cl, 4-F	35.89 ± 16.34	37.67 ± 1.61	23.99 ± 0.72
**26d**	3-CF_3_, 4-Cl	40.84 ± 8.23	48.26 ± 1.78	30.00 ± 1.14
**26e**	H	11.77 ± 0.50	20.77 ± 0.64	26.99 ± 4.95
**26f**	4-CH_3_	14.01 ± 1.32	9.33 ± 0.10	19.68 ± 1.96
**26g**	3-CH_3_, 4-CH_3_	29.82 ± 11.51	14.74 ± 0.03	16.87 ± 3.02
SAHA		93.34 ± 2.78	158.17 ± 6.66	78.98 ± 13.19

**Table 5:** HDAC inhibitory activity of compounds **35a**–**d**.

Compound	HDAC IC_50_ (μM)
**35a**	0.27 ± 0.004
**34b**	3.21 ± 0.10
**34c**	0.26 ± 0.05
**34d**	0.32 ± 0.05
SAHA	0.15 ± 0.02

**Table 6:** Inhibition of HDAC3 and HDAC8 isoforms by pyrazoles **61**, **67**, and **68a**–**d** compared with that of SAHA.

Compound	HDAC3 IC_50_ ± SD (nM)	HDAC8 IC_50_ ± SD (nM)
SAHA	27 ± 1.0	440 ± 21
**61**	128 ± 9.8	17 ± 3
**67**	432 ± 52	487 ± 80
**68a**	44 ± 5.8	76 ± 5.0
**68b**	59 ± 1.0	82 ± 9.0
**68c**	22 ± 1.3	28 ± 3.0
**68d**	191 ± 18	147 ± 15

**Table 7:** Biological activity of indazoles **16a**–**d** towards some HDAC isoforms and cancer cell lines.

Comp.	Alkyl Chain Linker *n*	HDAC1 IC_50_ (nM)	HDAC2 IC_50_ (nM)	HDAC8 IC_50_ (nM)	HCT-116 IC_50_ (μM)	MCF-7 IC_50_ (μM)	HeLa IC_50_ (μM)
SAHA		13	70	44			
**16a**	*n* = 3	76	168	54	>50	41.5	>50
**16b**	*n* = 4	13	62	41	23.5	4.4	5.8
**16c**	*n* = 5	2.6	6.3	4.5	10.6	7.4	20.1
**16d**	*n* = 6	1.9	3.9	3.0	4.9	0.8	5

## Data Availability

Not applicable.
